# Longitudinal Profiling of Antibody Response in Patients With COVID-19 in a Tertiary Care Hospital in Beijing, China

**DOI:** 10.3389/fimmu.2021.614436

**Published:** 2021-03-15

**Authors:** Xia Feng, Jiming Yin, Jiaying Zhang, Yaling Hu, Yabo Ouyang, Shubin Qiao, Hong Zhao, Tong Zhang, Xuemei Li, Lili Zhang, Jie Zhang, Ronghua Jin, Yingmei Feng, Bin Su

**Affiliations:** ^1^Beijing Youan Hospital, Capital Medical University, Beijing, China; ^2^Beijing Institute of Hepatology, Beijing Youan Hospital, Capital Medical University, Beijing, China; ^3^Sinovac Biotech Ltd., Beijing, China; ^4^Beijing Fengtai Hospital of Integrated Traditional and Western Medicine, Beijing, China; ^5^Department of Infectious Diseases, Peking University First Hospital, Beijing, China; ^6^Beijing Key Laboratory of Monoclonal Antibody Research and Development, Sino Biological Inc., Beijing, China

**Keywords:** COVID-19, SARS-CoV-2, spike, RBD, S antigen, neutralizing antibodies

## Abstract

The novel coronavirus named severe acute respiratory syndrome coronavirus 2 (SARS-CoV-2) caused a global pandemic of the coronavirus disease 2019 (COVID-19), which elicits a wide variety of symptoms, ranging from mild to severe, with the potential to lead to death. Although used as the standard method to screen patients for SARS-CoV-2 infection, real-time PCR has challenges in dealing with asymptomatic patients and those with an undetectable viral load. Serological tests are therefore considered potent diagnostic tools to complement real-time PCR-based diagnosis and are used for surveillance of seroprevalence in populations. However, the dynamics of the antibody response against SARS-CoV-2 currently remain to be investigated. Here, through analysis of plasma samples from 84 patients with COVID-19, we observed that the response of virus-specific antibodies against three important antigens, RBD, N and S, dynamically changed over time and reached a peak 5–8 weeks after the onset of symptoms. The antibody responses were irrespective of sex. Severe cases were found to have higher levels of antibody response, larger numbers of inflammatory cells and C-reactive protein levels. Within the mild/moderate cases, pairwise comparison indicated moderate association between anti-RBD vs. anti-N, anti-RBD vs. anti-S1S2, and anti-N vs. anti-S1S2. Furthermore, the majority of cases could achieve IgM and IgG seroconversion at 2 weeks since the disease onset. Analysis of neutralizing antibodies indicated that these responses were able to last for more than 112 days but decline significantly after the peak. In summary, our findings demonstrate the longitudinally dynamic changes in antibody responses against SARS-CoV-2, which can contribute to the knowledge of humoral immune response after SARS-CoV-2 infection and are informative for future development of vaccine and antibody-based therapies.

## Introduction

Coronavirus disease 2019 (COVID-19), caused by a novel coronavirus named severe acute respiratory syndrome coronavirus 2 (SARS-CoV-2), has affected over 190 countries and was declared a global public health concern (1, 2). Although extensive efforts have been made to reduce person-to-person transmission of COVID-19 and control the outbreak, the number of cases is still increasing according to the situation report by the World Health Organization (WHO) (3). Globally, on February 13, 2021, more than 107 million cases have been reported, including about 2.3 million deaths caused by the novel coronavirus (3, 4). At this time, Chinese mainland has confirmed 89,763 cases, including 4,636 deaths (5).

The current COVID-19 pandemic rapidly spread globally, making the development of effective countermeasures to cure and prevent this disease a major global priority. It is known that four structural proteins of SARS-CoV-2, spike surface glycoprotein (S), membrane protein (M), envelope protein (E) and nucleocapsid protein (N), are essential for coronavirus assembly and infection (6, 7). S protein is the best studied coronavirus protein. The S protein consists of S1 and S2 subunits, which mediate viral attachment to host cells and fusion, respectively, in the process of infection. To engage the receptor of the host cell, the receptor-binding domain (RBD) at the N-terminal of the S1 subunit undergoes hinge-like conformational alterations (8–12). N protein oligomerizes to form a closed capsule that wraps the genomic coronavirus RNA, providing the first-line defense from the harsh conditions of the host (13–15). S and N proteins are also known as the major immunogens for the antibody response against coronaviruses (10, 14, 16). S protein has epitopes recognized by T and B cells, which can induce the production of neutralizing antibodies (nAbs); therefore, S protein represents a target for antibody-mediated neutralization and diagnostics (17–21). N protein can also potentially induce humoral and T-cell immune responses and be logically chosen as a target antigen for vaccination (22–24).

Serologic assays are urgently required for tracing patient contact, identifying the viral reservoir and conducting epidemiologic studies, although molecular diagnostic tests were rapidly developed to support case identification and track the outbreak of the SARS-CoV-2 pandemic. The ways in which the antibody responds to SARS-CoV-2 remain poorly understood, and specific data on the response of humoral immunity during infection are still unclear (25). In this study, with plasma specimens collected from patients with COVID-19 in a tertiary care hospital in Beijing, we performed longitudinal profiling of IgM and IgG against SARS-CoV-2-neutralizing and RBD-, S1S2- and N-specific antibodies, which revealed the duration of the antiviral immune response and the dynamics of these antibodies during the epidemic outbreak of SARS-CoV-2.

## Methods

### Cohort Study

The COVID-19 case definition and clinical classification based on severity were defined according to the New Coronavirus Pneumonia Prevention and Control Protocol for COVID-19 (seventh edition) released by the National Health Commission of China (26). The clinical classification criteria were listed as follows. (1) Mild cases: clinical symptoms were mild without manifestation of pneumonia on imaging; (2) Moderate cases: fever, respiratory symptoms, and with radiological findings of pneumonia; (3) Severe cases: meeting any one of the following criteria: respiratory distress, hypoxia (SpO2 ≤ 93%), or abnormal blood gas analysis PaO_2_/FiO_2_ ≤ 300 mmHg, or who required mechanical ventilation either invasively or noninvasively (26). Eighty-four patients diagnosed with SARS-CoV-2 from Beijing Youan Hospital, China, from February 03 to May 18, 2020, were enrolled in this study (Table 1). Weekly followed-up and SARS-CoV-2 RNA detection were performed timely. The whole follow-up lasted for over 112 days and were divided into 7 time points since symptom onset. The time points were defined as the days after symptom onset in which samples were collected, from the first to seventh time points were days 1–7, 8–14, 15–28, 29–56, 57–84, 85–112, and >112, respectively. The throat swabs from the upper respiratory tract and whole blood were collected from patients at various time-points after hospitalization and during followed-up. Sample collection, processing, and laboratory testing were performed as recommended by China CDC and complied with WHO guidance. All COVID-19 patients were confirmed as infected based on positive results from their respiratory swab samples by RT-PCR tests. None of the study participants was co-infected with HIV, hepatitis B virus/hepatitis C virus, or influenza viruses. All participants do not have a comorbid condition, tuberculosis, autoimmune diseases, or related drug usage. In this study, 3 healthy individuals were recruited as controls, and the code “0” represents the healthy condition.

Table 1Demographic and clinical characteristics of COVID-19 patients.

**Table:** 

**Condition**	**Sex**	**Chronic respiratory disease**	**Coronary disease**	**Hypertension**	**Diabetes**	**Respiratory symptoms**
Mild/moderate cases	62	1	3	6	7	48
Female	36	0	2	2	3	32
Male	26	1	1	4	4	16
Severe cases	22	1	7	9	2	21
Female	14	1	4	5	2	13
Male	8	0	3	4	0	8
Total	84	2	10	15	9	69

**Table:** 

	**Mild/moderate cases**	**Severe cases**	***P*****-value**
Age (years)	44.84 ± 11.98	61.55 ± 14.76	<0.0001
Body temperature (°C)	38.01 ± 0.93	38.43 ± 0.83	0.0683
WBC (×10^9^/L)	4.46 ± 1.55	5.37 ± 1.95	0.0304
NEU (×10^9^/L)	2.69 ± 1.29	3.92 ± 2.08	0.0018
LYM (×10^9^/L)	1.32 ± 0.54	1.03 ± 0.49	0.0177
MNC (×10^9^/L)	0.32 ± 0.14	0.30 ± 0.14	0.0004
CRP (mg/L)	15.93 ± 21.56	54.27 ± 49.08	<0.0001

*All the data are expressed as mean ± standard deviation (SD) or n (%). The statistic analysis was performed using t-test, with p-values < 0.05 were considered significant. WBC, White blood cells; NEU, Neutrophils; LYM, Lymphocytes; MNC, Monocytes; CRP, C-Reactive Protein*.

### Ethics Statement

This study and part of the relevant experiments were approved by the Beijing Youan Hospital Research Ethics Committee (No. 2020-037) and written informed consent was obtained from each participant in accordance with the Declaration of Helsinki. The clinical samples were collected for research use. The methods used conformed to approved guidelines and regulations.

### Detection of the Antibodies Titer Against SARS-CoV-2

The titer of antibodies against structural protein RBD, N, and S1S2 were determined using indirect enzyme-linked immunosorbent assay (ELISA) kit supplied by Sino Biological Inc. (Cat: KIT004 for anti-S1S2 antibody; Cat: KIT005 for anti-N antibody; Cat: KIT006 for anti-RBD antibody, respectively). Briefly, corresponding recombinant proteins (RBD, N, and S1S2) have been pre-coated onto 96-well plates. The samples which were first diluted at 1:200 before measurement were added to the wells, followed by incubation with goat anti-human IgG conjugated with horseradish peroxidase for 1 h at 37°C after 5 washes with phosphate-buffered saline. The plates were developed using TMB, followed by 2 M sulfuric acid (H_2_SO_4_) addition to stop the reaction and colors developed in proportion to the amount of antibodies. To determine the final result, the ELISA plate was read at 450/630 nm by ELISA plate reader.

### Detection of the IgG and IgM Antibodies Against SARS-CoV-2

The IgG and IgM antibodies against SARS-CoV-2 in plasma samples were tested using Immune Capture Colloidal Gold kit (Lot: 20200303) supplied by Bioscience (Yingnuote) Co., Ltd., China (CFDA approved), according to the manufacturer's instructions.

### Neutralizing Antibody Titer Assay

The neutralizing antibody titers in plasma were measured by the Reed-Muench method on days 14 and 84 after discharge from the hospital. For calculation of geometric mean titer (GMT), antibody titers of <1:8, >1:512, and >1:1,024 were assigned values of 1:4, 1:(512 + 512/2), and 1:(1,024 + 1,024/2), respectively (27).

The titer of neutralizing antibody in plasma was determined with a modified cytopathogenic assay according to a previously published article (27). Briefly, plasma samples were inactivated at 56°C for 30 min and serially diluted with cell culture medium in 2-fold steps. The diluted plasma was mixed with a virus suspension of 100 CCID50 in 96-well plates at a ratio of 1:1, followed by 2 h incubation at 36.5°C in a 5% CO_2_ incubator. Then, 1 – 2 × 10^4^ Vero cells were added to the plasma-virus mixture, and the plates were incubated for 5 days at 36.5°C in a 5% CO_2_ incubator. The cytopathic effect (CPE) of each well was recorded under microscopes, and the neutralizing titer was assayed by the dilution number of the 50% protective condition.

### Statistical Analysis

Statistical difference was analyzed using Student's *t*-tests or Wilcoxon test with GraphPad Prism software version 5.03 (GraphPad Software, San Diego, California, USA). Differences were considered statistically significant at *p* < 0.05.

## Results

### Demographic and Clinical Characteristics

A total of 84 patients with COVID-19 were enrolled in this study, including 34 males and 50 females. The average age of the patients was 48.85 ± 13.26 years for males and 49.48 ± 15.68 years for females (data is not shown in the Table 1). The clinical data for patients' disease history such as chronic respiratory disease, coronary disease, hypertension, and diabetes were summarized in Table 1 (upper panel). Comparison analysis revealed that severe cases had higher levels of inflammatory cells and C-reactive protein (Table 1, down panel).

### Enhanced Response of Antibodies Against SARS-CoV-2 Structural Proteins

**Figure 1:**
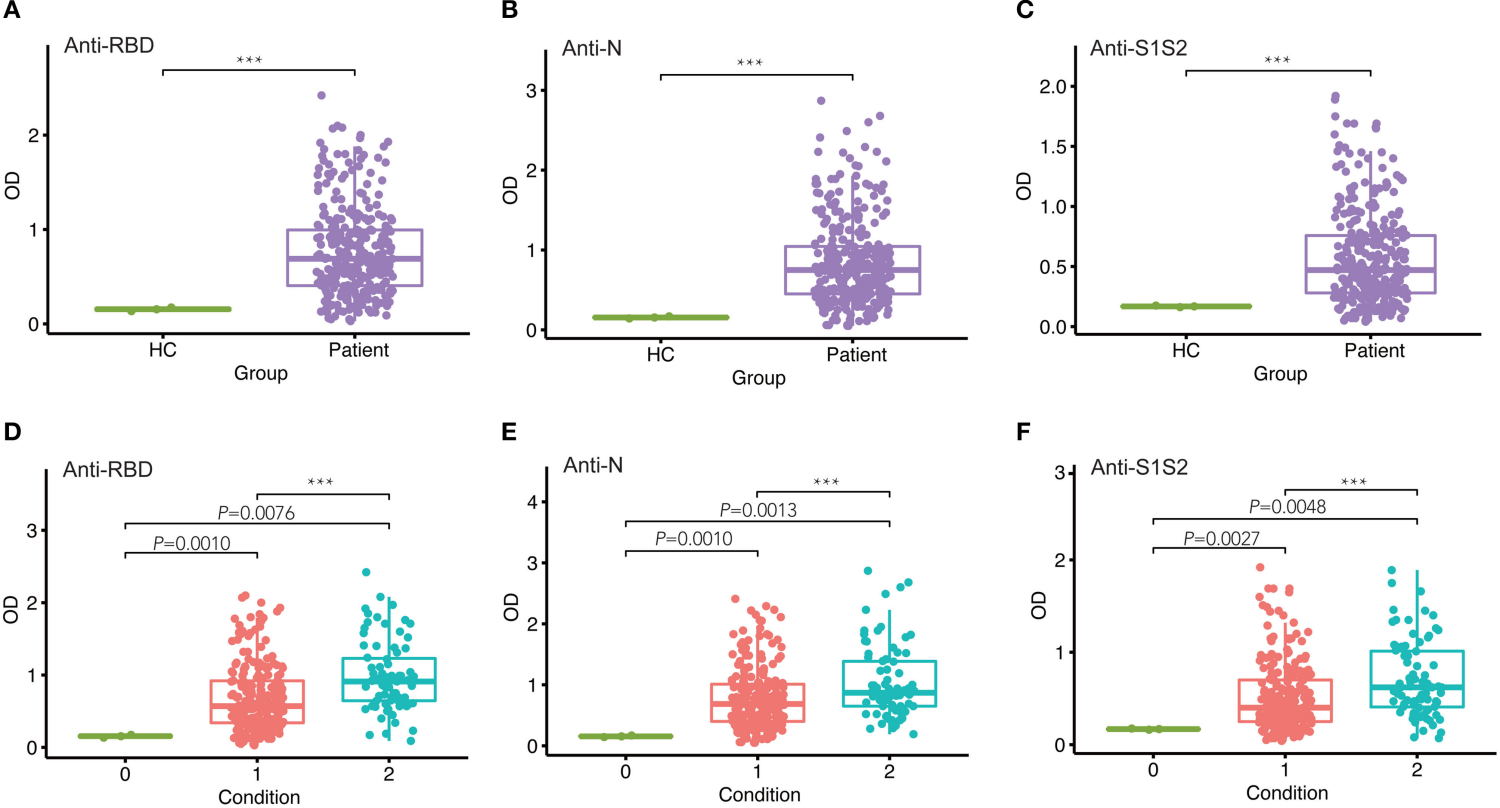
Comparison of antibody responses between different groups. **(A–C)** Comparison of anti-RBD **(A)**, anti-N **(B)**, and anti-S1S2 **(C)** antibody responses between healthy controls (HC) and infected patients. **(D–F)** Pairwise comparison of anti-RBD **(D)**, anti-N **(E)**, and anti-S1S2 **(F)** antibody responses between healthy (“0”), mild/moderate (“1”), and severe (“2”) conditions. *P*-values were calculated by Student's *t*-test for **(A–C)** and by one-way ANOVA for **(D–F)**. *P*-values < 0.05 were considered significant, and labeled with ***P < 0.001.

To assess the response of antibodies against RBD, N, and S1S2, we performed in-house ELISAs to detect their presence in patients throughout the study. First, we compared the levels of these antibodies in patients with those in healthy individuals and observed that they were all markedly increased in patients (*p* < 0.001, Figures 1A–C). Furthermore, we classified the patients into two groups according to the severity of COVID-19 at admission and performed pairwise comparisons. For each type of antibody, we also observed that the levels were significantly higher in COVID-19 patients than in healthy controls, and this rising trend was maintained from mild/moderate conditions to severe conditions (Figures 1D–F).

### Dynamics of Antibody Levels With Progression and Severity of Disease

**Figure 2:**
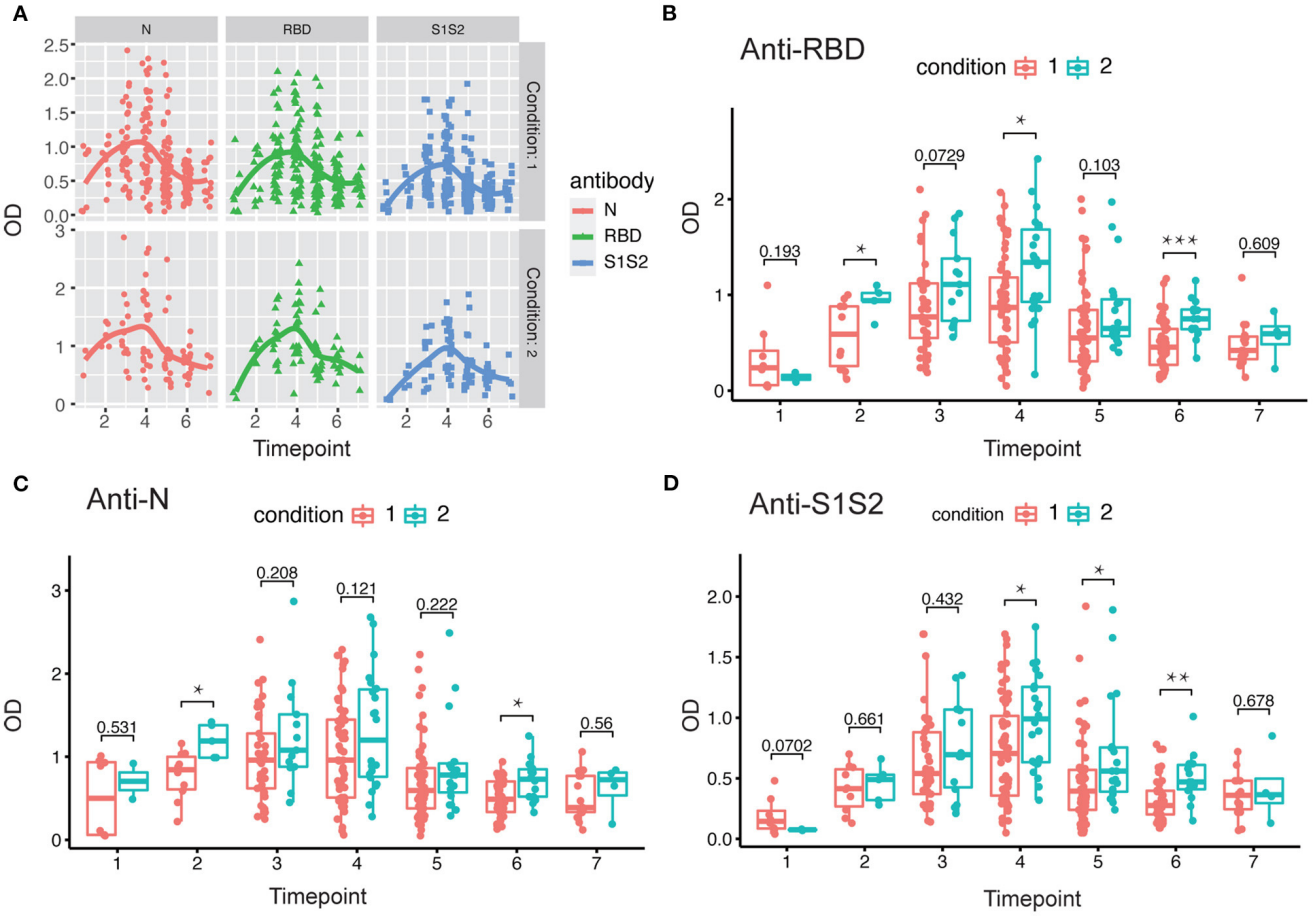
Evaluation of antibody response between mild/moderate and severe patients. **(A)** Response of antibodies (anti-N, anti-RBD, and anti-S1S2) between mild/moderate and severe patients. **(B–D)** Comparison of anti-RBD **(B)**, anti-N **(C)**, and anti-S1S2 **(D)** antibody responses between mild/moderate (“1”) and severe (“2”) patients. Time points 1–7 represent days 1–7, 8–14, 15–28, 29–56, 57–84, 85–112, and >112, respectively, after symptom onset. *P*-values were calculated by Student's *t*-test. *P*-values < 0.05 were considered significant with *, **, and *** indicate *p* < 0.05, <0.01, and <0.001, respectively.

Next, we investigated the dynamics of antibody levels throughout the study period. The observations in the period showed that the levels of anti-RBD, anti-N, and anti-S1S2 increased over time and peaked around at the fourth time point and then decreased slowly. These changing patterns were similar among patients in both mild/moderate and severe conditions (Figure 2A). Moreover, the levels of antibodies, including anti-RBD (Figure 2B), anti-N (Figure 2C), and anti-S1S2 (Figure 2D), in patients with a severe condition were relatively higher than those in patients with a mild/moderate condition. These differences were prominent in the late stage, especially for the anti-S1S2 antibody from the fourth to sixth time points.

**Figure 3:**
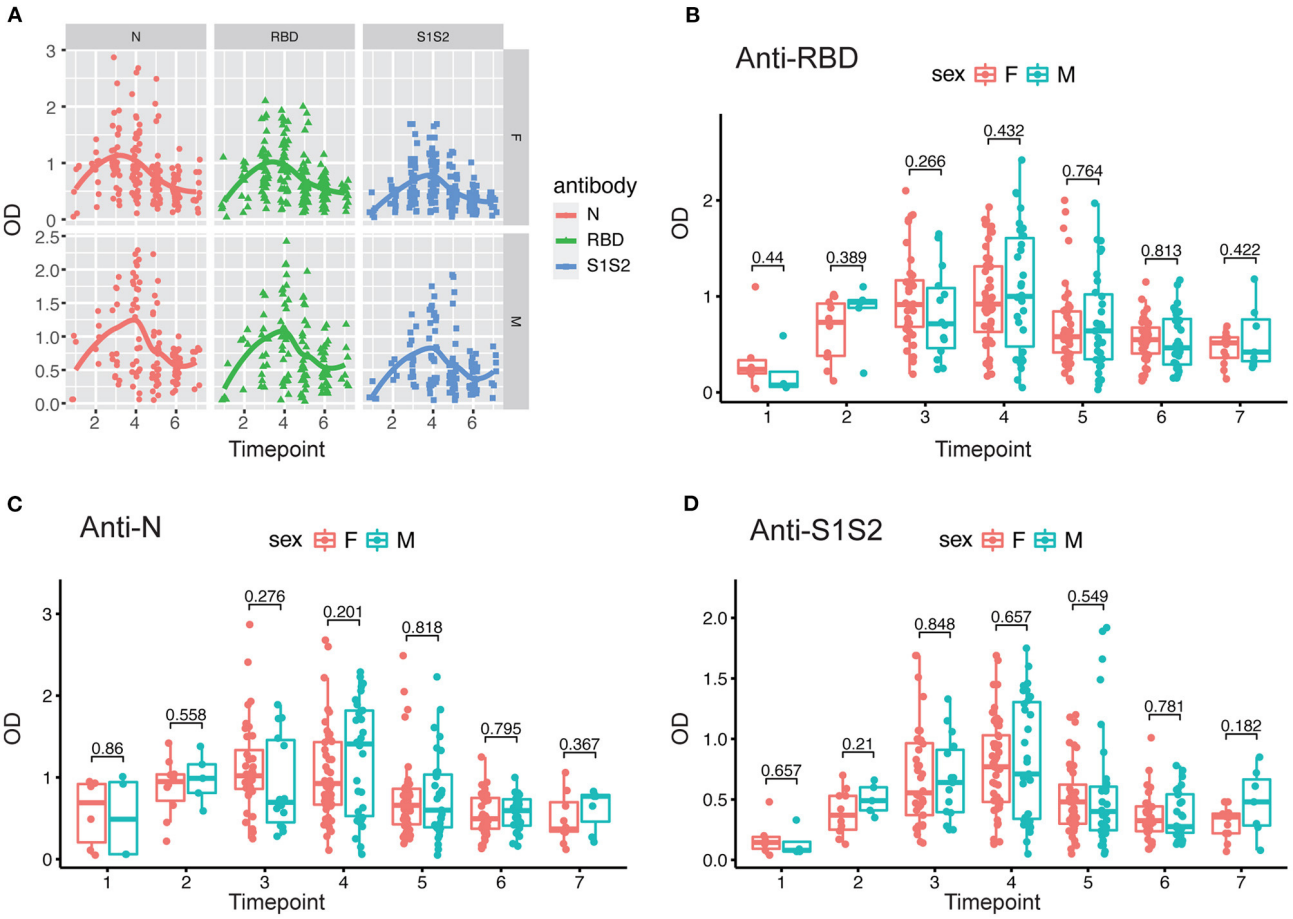
Evaluation of antibody response between female and male patients. **(A)** Response of antibodies (anti-N, anti-RBD, and anti-S1S2) between female and male patients. **(B–D)** Comparison of anti-RBD **(B)**, anti-N **(C)**, and anti-S2S2 **(D)** antibody responses between female (“F”) and male (“M”) patients. Time points 1–7 represent days 1–7, 8–14, 15–28, 29–56, 57–84, 85–112, and >112, respectively, after symptom onset. *P*-values were calculated by Student's *t*-test, and *p*-values < 0.05 were considered significant.

In addition, we performed the same comparisons for the antibody levels between female and male patients. The dynamics of antibodies against N, RBD, and S1S2 proteins displayed similar wave patterns as those shown in previous analyses (Figures 2A, 3A). Moreover, for the three types of antibodies at each time point, there were no differences between female and male patients (Figures 3B–D). These findings suggest that the levels of antibodies against the main SARS-CoV-2 immunogenic proteins are related to the progression and severity of COVID-19.

**Figure 4:**
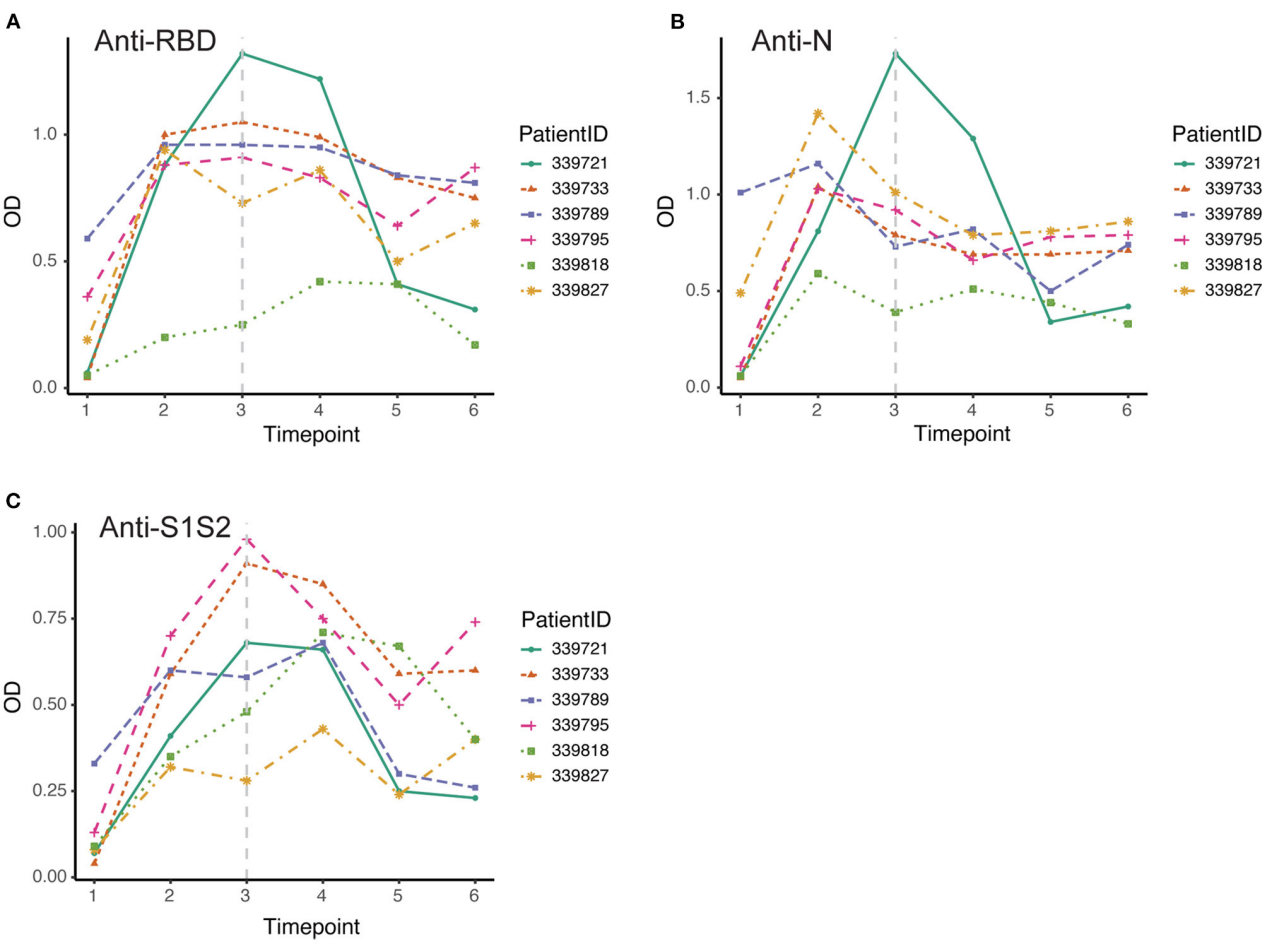
Dynamic changes in antibodies against SARS-CoV-2 structural proteins. **(A–C)** Dynamic changes in anti-RBD antibody **(A)**, anti-N antibody **(B)** and anti-S1S2 **(C)** in representative patients over the monitoring period. Time points 1–6 represent days 1–7, 8–14, 15–28, 29–56, 57–84, and 85–112, respectively, after symptom onset. The gray vertical dashed line indicates the approximate time of discharge.

Furthermore, we screened 6 patients who had good follow-up compliance and observed their antibody responses individually. We collected plasma samples from these patients at 6 time points (first through the sixth). By the discharge time at the third time point, all patients except one exhibited the peak of the anti-RBD antibody (Figure 4A). In contrast, all patients achieved the peak response of anti-N antibody by that time (Figure 4B), and for the response of anti-S1S2 antibody, we observed that the peak response in three patients lagged until the fourth time point. Moreover, two patients had a rebounded response to anti-S1S2 antibody at the sixth time point (Figure 4C).

### Dynamics of Antibody Seroconversion in Patients Since Symptom Onset

**Figure 5:**
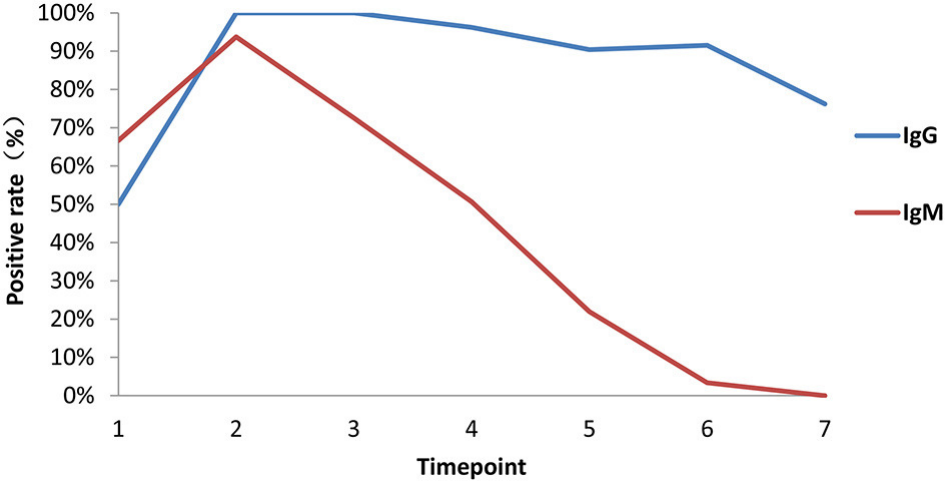
Proportions of patients with seroconversion of IgM and IgG changes. Positive rates of virus-specific IgG (blue) and IgM (red) at different times in 84 plasma samples. Time points 1–7 represent days 1–7, 8–14, 15–28, 29–56, 57–84, 85–112, and >112, respectively, after symptom onset.

Among 84 patients, 66.7% (56/84) were positive for virus-specific IgM during the 4-month observation period. One patient was found to be positive for IgM on the day of symptom onset, and the last positive detection among the remaining patients who had IgM positive conversion was on the sixteenth day after symptom onset. Among IgM-positive patients, 57.1% (48/56) finally underwent IgM seronegative conversion, which occurred as early as day 32 after symptom onset. The median day of seronegative conversion for IgM was 57 (range: 19–82 days) after symptom onset. Eighty-four patients were all positive for virus-specific IgG, which became detectable as early as day 3 and as late as day 16 after symptom onset. However, 15.5% (13/84) underwent seronegative conversion, which occurred as early as day 51 after symptom onset. Among these patients with seronegative conversion, 92.3% (12/13) had a mild/moderate condition, and 7.7% (1/13) were had a critical condition. The negative seroconversion for IgG did not display a significant difference between mild/moderate and severe patients (*p* > 0.05, χ^2^ = 2.722). Overall, the longitudinal changes in virus-specific IgM and IgG antibodies in all patients are shown in Figure 5. The proportion of patients with detectable IgM reached 67% at the first time point (day 1–7 after onset), while the proportions of patients with detectable IgG were almost equal to 50% in the same monitoring period. The proportions of IgM- and IgG-positive patients reached 94 and 100%, respectively, at the peak in the second time point. Subsequently, the proportion of IgM-positive patients showed a rapid decrease over time, while the proportion of IgG-positive patients maintained relatively stable. Only from the fourth time point (day 29–56) did the proportion of IgG-positive patients decrease slightly.

### Decline in Neutralizing Activities Over Time

**Figure 6:**
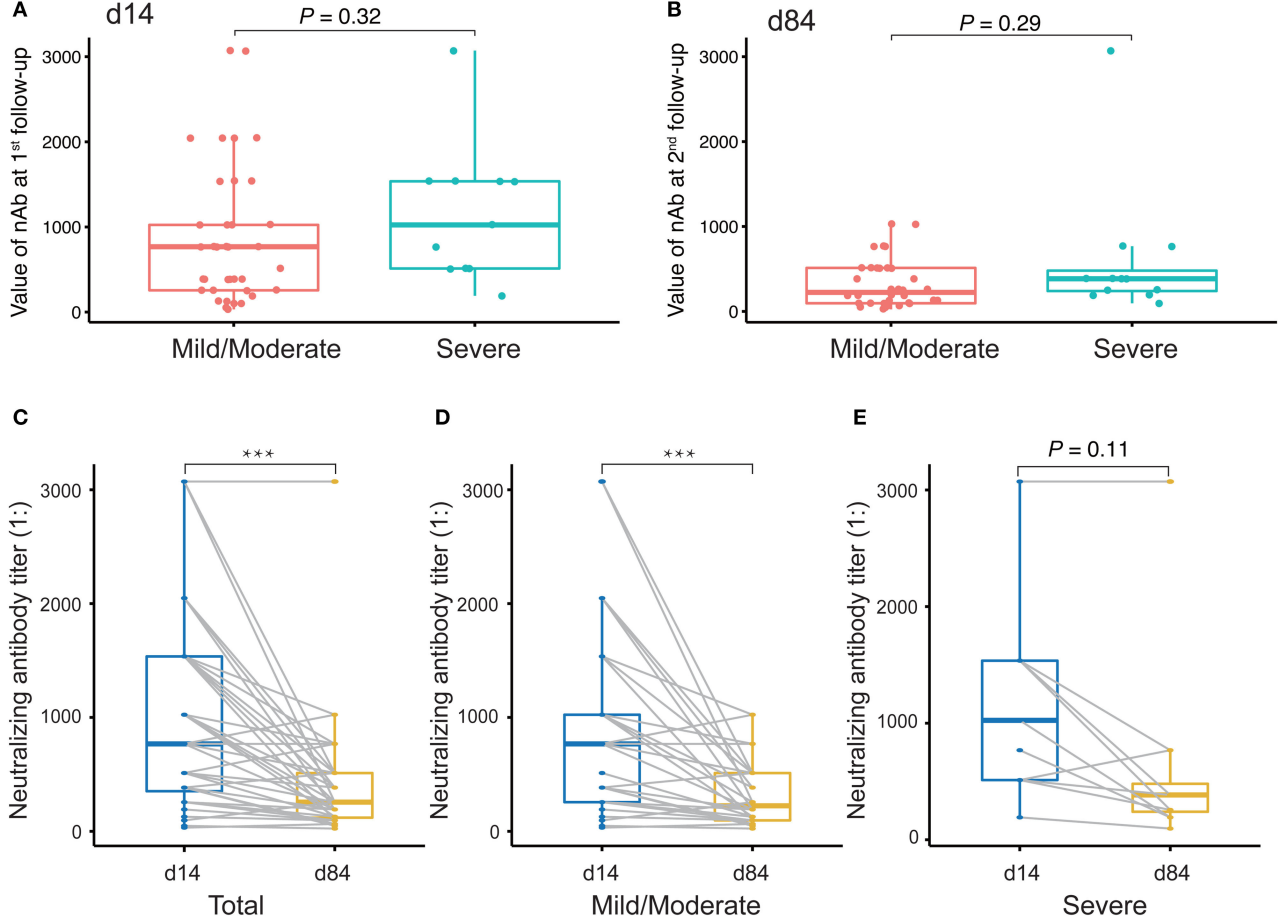
Evaluation of neutralizing antibody (nAb) levels in COVID-19 patients discharged. **(A,B)** Unpaired *t*-test analysis to compare nAb levels in COVID-19 patients discharged between mild/moderate condition and severe condition at the first follow-up (at day 14 after discharge, **A**) and at the second follow-up (at day 84 after discharge, **B**). **(C–E)** Paired *t*-test analysis to compare nAb levels in patients between the first and the second follow-ups for both mild/moderate and severe conditions **(C)** as well as for each conditions, mild/moderate **(D)** and severe **(E)**, respectively. *P*-values < 0.05 were considered significant and labeled with ***P < 0.001.

Finally, we evaluated changes in the levels of nAbs against SARS-CoV-2 by measuring the titers of nAbs in 49 plasma samples collected at the first time point and the last time point during the monitoring period after discharge. The interval between two sampling times was approximately 2 months. The first sampling took place around the third or fourth time points (14 days after discharge), and the second sampling took place around the sixth time point (84 days after discharge). Initially, we compared the levels of nAbs between patients with a mild/moderate condition and severe condition. The results showed that there was no difference between these conditions either at the first follow-up monitoring at day 14 (Figure 6A) or the second follow-up monitoring at day 84 after discharge (Figure 6B). However, we performed a paired comparison for the levels of nAbs between the two follow-ups and found that there was an obvious decline in nAb levels over the period (Figure 6C). Furthermore, the remarkable change of nAb levels was found to maintain in mild/moderate patients (Figure 6D) instead of severe ones (Figure 6E), suggesting that the response of nAbs has to do with the disease conditions. Taken together, our study highlights the need for prospective serology studies assurance to better understand the humoral response to SARS-CoV-2 infection.

## Discussion

In this study, we aimed to assess SARS-CoV-2 seroprevalence in patients hospitalized in a tertiary care hospital in Beijing as well as to evaluate longitudinal changes in antibody levels within the first 4 months after onset of disease and how the changes correlate with sex and COVID-19 symptoms. We tested plasma samples and monitored the dynamic changes in anti-RBD, anti-N, and anti-S1S2 antibodies in 84 COVID-19 patients (62 mild/moderate cases and 22 severe cases). Our results showed that antibodies were elicited against RBD, N, and S1S2 of SARS-CoV-2 over time, and in general, all the antibodies reached a peak 5–8 weeks after symptom onset. The three antibodies presented similar profiles regardless of patient condition and sex. The duration of antiviral antibodies could last for more than 112 days. However, the levels of antibodies (anti-RBD, anti-N, and anti-S1S2) were significantly higher in patients with a severe condition (Figures 1–3). Another difference was that anti-RBD and anti-N antibodies appeared to synchronously achieve seroconversion and reached a peak approximately 2–3 weeks after onset, while anti-S1S2 antibodies in some patients reached a peak more than 4 weeks after onset (Figure 4). In addition, we assessed the correlations among the anti-RBD, anti-N, and anti-S1S2 antibody levels at the discharge time point, and they exhibited moderate relationships with each other (anti-RBD vs. anti-N, *r* = 0.76; anti-RBD vs. anti-S1S2, *r* = 0.83; and anti-N vs. anti-S1S2, *r* = 0.73, respectively). We further performed multiple regression analyses with condition, sex and age as independent covariates and the antibody levels as the response; this analysis showed that none of these factors were significant (data not shown), probably due to small sample sizes.

In addition, the proportion of IgM seropositive conversion was higher than that of IgG the first week after symptom onset, which is consistent with the wide recognition that IgM provides the first line of defense during viral infection. Accordingly, IgM detection in the plasma also revealed the proportion of patients who had a recent exposure to SARS-CoV-2, while IgG detection suggested that the exposure happened several days before and in the recovery phase. However, in most cases, it is difficult to accurately determine the exact time when a patient contracted the virus. The proportion of IgM conversion increased to the highest level (94%) 2 weeks after symptom onset, while that of IgG conversion reached 100% at the same time point. After the peak, both IgM and IgG declined, but IgM dropped more rapidly over time (Figure 5). Moreover, nAbs also showed a significant decline, reflecting the recovery of disease and clearance of viruses (Figures 5, 6), consistent with other studies (28, 29). However, in our study, among the 49 samples of neutralizing activities, 37 cases decreased, 4 cases unchanged, and 8 cases increased, suggesting that the dynamic change of neutralizing responses varied greatly in individuals, but it tended to decline as a whole. Further studies are required to investigate this mechanism. Taken together, our findings raise concern that humoral immunity against SARS-CoV-2 may not be long lasting in persons with mild/moderate illness, who compose the majority of persons with COVID-19 (28).

Serologic assays are crucial for patient contact tracing and epidemiologic studies. Since the critical components of SARS-CoV-2, S and N proteins, are essential for the mediation of viral infection, they have already received much attention in studies on induced antibody responses (19, 20, 30–38). As described in previous studies (39), our findings showed that the humoral immune response could be maintained for approximately 4 months (>112 days). However, almost all reports are based on a limited number of cases due to the urgent situation. These reports also have some discrepancies between each other with regard to the time of antibody response or seroconversion or the levels of antibodies or the proportions of seroconversion, probably because there is a lack of uniform standards for enrollment of study subjects or in-house assay development. Therefore, it is necessary to gather more information on the antibody response during the process of COVID-19 infection to provide more comprehensive and accurate knowledge. For example, in our study, only 66.7% of cases (56/84) showed positive IgM conversion, while Zhao et al. reported a ratio up to 82.7% (143/173) (32). Moreover, studying antibody responses can provide important clues for the development of vaccines and therapeutic antibodies for the prevention of the disease. S and N proteins, as critical immunogens, have been proposed to have the most clinically relevant value to provide protection against coronaviral infection. Recently, animal experiments demonstrated that the spike protein of SARS-CoV-2 can trigger strong protective antibody responses in rabbits (16). In addition, the combination of subunit vaccines with appropriate adjuvants may provide a good strategy for early clinical development (40).

Like others, our study has some limitations. First, due to the small sample size of patients with severe conditions, it is difficult to draw conclusions on the relationship between antibody response and clinical course. Second, because of the limited number of longitudinal plasma samples from the patients, it was also difficult to accurately assess the seroconversion time. Consequently, if the levels of antibodies are not high enough at the time of measurement, false negatives could be recorded. Therefore, a larger number of longitudinal samples is needed. Indeed, further investigation is needed to determine the reasons for negative IgM results in patients (28/84), and asymptomatic patients with an undetectable viral load. Previous studies showed possible reasons, including extreme low viral load and insufficient sensitivity of kit detection, limitation of specimen types and irregular collection and intermittent virus shedding (41). Nevertheless, our findings contribute additional clinical information to the knowledge of antibody response during SARS-CoV-2 infection. As virological detection of SARS-CoV-2 through RT-PCR has limitations for surveillance, serological tests can detect if a person has been infected even months after viral clearance, which with very high sensitivity and specificity can be an important complementary approach (42, 43).

Taken together, our findings can benefit local researchers estimating the extent of the spread of the COVID-19 pandemic, provide comprehensive information on kinetics and neutralizing antibody responses in COVID-19 patients, and will improve our understanding for the development of vaccines as well as shed light on diagnosis, prognosis, and other treatments of SARS-CoV-2 infection (44).

## Data Availability Statement

The original contributions presented in the study are included in the article/supplementary material, further inquiries can be directed to the corresponding author/s.

## Ethics Statement

The studies involving human participants were reviewed and approved by the Beijing Youan Hospital Research Ethics Committee (No. 2020-037) and written informed consent was obtained from each participant in accordance with the Declaration of Helsinki. The clinical samples were collected for research use. The methods used conformed to approved guidelines and regulations.

## Author Contributions

BS, YF, and RJ conceived the study, designed the experiments, and analyzed the data. XF, JY, JiaZ, YH, YO, SQ, HZ, XL, LZ, and JieZ performed the experiments, carried out the data collection and data analysis. TZ, RJ, and YF contributed to reagents and materials. XF, JY, and BS wrote the article and revised the manuscript. BS supervised the manuscript. All authors read and approved the final manuscript.

## Conflict of Interest

YH was employed by Sinovac Biotech Ltd. JZ was employed by Sino Biological Inc. The remaining authors declare that the research was conducted in the absence of any commercial or financial relationships that could be construed as a potential conflict of interest.
